# Revolutionizing medical physiology education: a narrative review of the transition from traditional classrooms to artificial intelligence

**DOI:** 10.3389/fmed.2026.1781197

**Published:** 2026-05-26

**Authors:** Adham Al-Rahbi, Harshita Agarwal, Srinivasa Rao Sirasanagandla, Mohammad Orooj Azmi, Qusay Al-Badi, Aditi Kolekar, Srinivas Rachoori, Raju Suresh Kumar, Hussein Sakr, Mickael Antoine Joseph

**Affiliations:** 1 Oman Medical Specialty Board, Muscat, Oman; 2 Institute of Post Graduate Medical Education and Research, Kolkata, India; 3 Department of Human and Clinical Anatomy, College of Medicine and Health Sciences, Sultan Qaboos University, Muscat, Oman; 4 College of Nursing, Sultan Qaboos University, Muscat, Oman; 5 Government Medical College Alibag, Raigadt, India; 6 Government Medical College, Omandurar Government Estate, Chennai, India; 7 Division of Basic Medical Sciences, College of Medicine, Dhofar University, Salalah, Oman; 8 Physiology Department, College of Medicine and Health Sciences, Sultan Qaboos University, Muscat, Oman; 9 Fundamentals and Administration Department, College of Nursing, Sultan Qaboos University, Muscat, Oman

**Keywords:** artificial intelligence, gamification, medical education, personalized learning, simulation

## Abstract

Medical physiology is a foundational component of medical education, offering vital details about normal human function and a deep understanding of disease processes. Traditionally taught through lectures, textbooks, and laboratory demonstrations, physiology education has progressively evolved in response to advances in technology and changing learner needs. This review examines the historical development of physiology teaching and synthesizes contemporary innovations that are transforming the educational landscape. Emphasis is placed on simulation-based education, hybrid and virtual classrooms, immersive technologies such as virtual and augmented reality, gamification, and student-centered learning strategies, all of which promote active engagement, conceptual understanding, and integration of physiological knowledge into clinical contexts. The review also explores the emerging role of artificial intelligence in physiology education, including personalized learning pathways, adaptive assessments, and real-time physiological simulations, with relevance for resource-limited settings. Innovations in assessment, competency-based medical education, interdisciplinary integration, and clinically oriented physiology teaching are highlighted as key contributors to improved learning outcomes. Additionally, the review addresses inclusivity and accessibility through the use of open educational resources and digital platforms that reduce disparities in educational access. Despite these advances, several challenges remain, including infrastructure limitations, faculty training gaps, institutional resistance, and ethical considerations surrounding the use of advanced technologies. Furthermore, the review outlines future directions focused on holistic learning environments, deeper clinical integration, and a balanced adoption of technology alongside traditional teaching methods. Overall, this narrative review highlights the importance of adopting a learner-centered, adaptable, and inclusive approach to physiology education that equips medical students for complex clinical practice and lifelong learning.

## 1 Introduction

### 1.1 History of physiology teaching

The history of physiology and anatomy cannot be separated from each other (1). In 1628, the science of physiology began with the publication of a book titled “An anatomical exercise on the motion of the heart and blood in living beings” by Harvey (1578–1657) (1). Physiology teaching began with the use of textbooks, lectures, and laboratory activities, focusing on the explanation of normal body function and homeostasis (2). An increased use of images or figures in physiology textbooks was only seen after the 1980s (3). Initially, the medical illustrations began to appear in black and white and then transitioned to multicolored in the 19th century (3). Later, advances in medical illustrations have revolutionized physiology education by simplifying complex concepts and enhancing student comprehension (4). The live experimental animals were used as complementary teaching material to demonstrate physiological principles. Additionally, students use each other as subjects, especially for measuring vitals, lung capacity, and other non-invasive physiological tests. Over time, physiology education has become more organized, relying on a structured curriculum and materials. The books employ videos on a CD-ROM and even computer software. Additionally, the advances of the 20th century impacted various educational fields, and physiology is one of them, as it began to integrate emerging Artificial Intelligence (AI) technologies as one of the pedagogy tools. The current state of AI in medical physiology education has been discussed in detail in this review.

### 1.2 Significance of physiology in medical education

Physiology is a subfield of biology that focuses on the function of living organisms, including respiration, digestion, reproduction, and nutrition (4). The co-author of Guyton and Hall’s Textbook of Medical Physiology, Dr. John E. Hall, stated that every year, the Association of American Medical Colleges (AAMC) found that physiology is the most important science in preparing medical students for clinical training, based on the students’ opinions (5). Physiology is a prerequisite and fundamental course for health-related sciences. Its comprehension is essential for understanding pharmacology, cellular biology, and pathophysiology of certain diseases (6). Physiology complements the understanding of human structure and anatomy by enabling learners to gain basic knowledge about the functionality of each element inside the human body. Moreover, understanding physiology is a cornerstone for better clinical practice. Physiology interacts with medicine in two ways. Physiological concepts mainly provide essential insight into medical practice (7). The other interaction is that medical knowledge and practice refine physiological concepts, as medical observations can lead to new insights into physiological processes. These data provide a valuable source for advances in physiological research and discoveries (7).

### 1.3 The need for evolving teaching strategies

Advances in physiology instruction must maintain the quality despite the increasing student enrollment capacity (6). Additionally, it should promote critical thinking to avoid rote memorization (6). The COVID-19 pandemic has accelerated the shift to remote laboratories and virtual classrooms (8). There are four major reasons to shift to virtual classrooms and laboratories (8). First, the physical laboratories are costly due to the high-end instruments and or equipment, and the expense of recruiting expert professors. Second, restrictions in experiments that are demonstrated on animals are due to ethical dilemmas associated with their use in experimentation. Third, virtual laboratories are much safer than physical ones. Finally, accumulated evidence from various scientific fields supports the integration of traditional methods with new virtual classrooms, as they show promising outcomes (8). However, for conceptually demanding topics such as physiology, fully remote instruction can reduce opportunities for immediate interaction, hands-on practice, and kinesthetic learning. Accordingly, we emphasize blended approaches. Hybrid physiology teaching methods that combine both online and physical classrooms represent an increasingly prevalent pedagogical model (6). It has been demonstrated that pre-post changes in mean exam scores were significantly greater in online classrooms. Moreover, the students’ satisfaction was higher in online methods (6). According to Anderson and Krichbaum, to promote knowledge acquisition and critical thinking, educational institutions should adopt new methods to enhance students’ learning, regardless of the medium (online or physical) (6).

### 1.4 Objective and scope of the review

This narrative review aims to explore the evolution of physiology teaching and to acknowledge the recent advances in the field. It focuses on innovative techniques and methodologies in teaching and learning. Additionally, this review examines recent trends and emerging challenges that hinder the adoption of these new strategies, as well as possible solutions. The recent recommendations for enhanced knowledge comprehension and active learning are also included in this review. The scope of this review encompasses hybrid classes, simulation-based education, Virtual Reality (VR) applications in teaching physiology, gamification, Artificial Intelligence AI, challenges, and future directions. To the best of the author’s knowledge, currently, there is limited literature that comprehensively reviews the strategies used and recent advances in physiology teaching, along with their limitations and solutions for better global adoption.

### 1.5 Methodology

To collect the data for this narrative review, we performed a comprehensive literature search in the electronic databases such as Scopus, Google Scholar, and PubMed. In addition, the reference lists of selected articles were manually screened to identify further relevant studies. Additional articles were also identified through citation tracking. All materials published before 30 September 2025 were included in this review. The keywords used to retrieve the relevant published data include “physiology,” “learning,” “education,” “teaching,” “simulation,” “gamification,” “artificial intelligence,” “machine learning,” “neural networks,” and “deep learning.” The inclusion criteria included all full-text articles written in English and covering AI technology application in physiology teaching for health science undergraduate students. The exclusion criteria included letters to the editor, abstract-only articles, commentary, non-human authors, and non-English articles.

## 2 Integration of technology in physiology education

Integrating technology to improve the current modus operandi is essential, largely due to the COVID-19 pandemic, which also led to rapid and large-scale adoption of e-learning and digital resources in medical education because of widespread lockdowns, social distancing measures, and the closure of medical schools and healthcare training programs (9). This transition is supported by the recent rise in e-learning platforms, web-based learning, and the maintenance of professional education quality through Massive Open Online Courses (MOOCs) (10). MOOCs utilize conventional directed instruction within the context of formal postsecondary educational institutions (10). There are three characteristics associated with MOOCs, namely varied definitions of openness, barriers to persistence, and a distinct structure through one of two pedagogical approaches (10). Research has focused on engagement, motivation, and presence to mitigate the risks of learner isolation.

Table 1 presents a summary of technology-based interventions in medical physiology education.

**Table 1:** Summary of technology-based interventions in medical physiology education.

References	Key findings	Methodology	Comparison	Strengths	Research gaps
(17)	Post-intervention understanding improved: 79.67% auditory masking, 91.05% lateralization (*p* < 0.001). 96.74% found the session engaging.	Pre-post interventional study (123 Medical students). Simulation of unilateral hearing loss using finger occlusion in tuning fork tests.	Compared understanding and engagement before vs. after the simulation	Simple, reproducible, cost-effective. Highly engaging.	No long-term retention or multi-institutional data.
(14)	Enhanced modeling of cardiovascular and respiratory functions. Allowed comparison of normal and abnormal states	Tools: JustPhysiology (acute sim), Sycamore (chronic care sim). Scenario-based physiological simulations.	Compared model fidelity, real-time feedback, and integration into the curriculum.	Interactive, clinically realistic	Requires digital infrastructure and tech support
(15)	Increased learner confidence in linking theory to practice.	Scenario-based simulation using vital signs, patient histories, and decision-making modules.	Applied physiology knowledge in the context of patient care.	Encourages clinical integration.	Primarily focused on nursing theories, less on core physiology.
(31)	ARS enhanced student engagement, knowledge retention, and academic performance, leading to significantly improved scores. Students found ARS easy to use and helpful.	Quasi-experimental study across ten lectures (five with ARS) for Health Sciences and pre-med students, comparing quiz scores and surveys. Feedback was collected.	Lectures with ARS vs. lectures without ARS	Easy to use, promotes engagement, active participation, and instant and anonymous feedback	Increased practice on how to integrate ARS into classrooms could lead to further utilization
(21)	HMDs (VR/AR) were perceived as engaging and useful for medical learning.	Systematic review of AR/VR headsets in medical education.	VR vs. traditional platforms in terms of learner engagement and outcome.	High interactivity and learner motivation.	VR primarily adds value as a supplement, not a full replacement for traditional teaching
(20)	VR increased learner interactivity, engagement, and retention across physiology topics.	Experimental study with VR modules across multiple physiological systems.	Compared traditional vs. VR-enhanced teaching	Improves interest and real-time comprehension.	Equipment access and cost may hinder wide adoption.
(24)	Significant improvement in cardiovascular physiology scores and engagement after game-based intervention.	Pre- and post-test study on 150 students using custom board/card games (CARBGAME).	Traditional lecture vs. gamified format.	Highly creative, experiential, and fun.	Short-term study; needs diverse topics and long-term follow-up.
(28)	The gamified group showed higher EEG-measured attention and test performance	Experimental study using EEG in gamified vs. non-gamified groups.	Physiological and performance data compared.	Objective measure of engagement.	Small sample (14 students); preliminary findings.
(22)	AI personalized learning improved engagement, motivation, and communication.	Evaluation of AI-powered modules for individual learning paths.	Compared AI-tailored vs. standard modules.	High accessibility, improved feedback loop.	Validating this approach is crucial, and assessing the faculty’s preparedness to overcome this challenge is essential.
(73)	Provided real-time physiological simulations; supported adaptive learning.	AI-powered platforms for simulating experiments and customizing difficulty levels.	Compared to textbook learning, visualization and self-paced exploration.	Widens access in under-resourced areas.	Technical challenges and costs for institutions.

### 2.1 Simulation-based education

Simulation-based education is a learner-centered strategy that utilizes realistic scenarios or environments to replicate clinical situations and physiological processes, thereby enabling students to practice and refine their skills in a safe and controlled setting (11). Simulation is rooted in the basic physiology of learning and the functioning of the human brain, which facilitates deeper comprehension of the subject (12). It addresses the limitations of purely didactic methods by allowing repeated practice, immediate feedback, and the ability to customize learning experiences (13). In physiology education, simulation is used to demonstrate complex physiological concepts. Hester and colleagues utilized simulation to teach cardiovascular hemodynamics and respiratory mechanics, enabling the modeling of both normal and abnormal physiology (14). This provided students with real-time visualization of how variables such as blood pressure respond to interventions. Moreover, simulation fosters the integration of physiological knowledge into clinical decision-making. With the addition of scenario-based patient charts, vital signs, and histories, students learn how underlying physiological mechanisms lead to certain pathologies or responses to treatments. By recreating real-life clinical conditions, simulation helps students apply theoretical knowledge (e.g., physiology concepts) directly to patient-care scenarios (15). This can take place in a safe and risk-free environment as errors in the simulation laboratory do not endanger actual patients, which fosters deeper learning. Students can repeat high-risk scenarios (e.g., acute life support) until they achieve proficiency (16). A recent study by Kumar and colleagues implemented a simulation-based intervention to enhance understanding of auditory physiology among first-year medical students. Using tuning fork tests and a simple technique to simulate unilateral conductive hearing loss by occluding the external auditory canal, students actively experienced auditory masking and sound lateralization. The intervention significantly improved conceptual understanding and engagement, demonstrating the effectiveness of hands-on simulation in medical education (17).

#### 2.1.1 Simulation types

Normal simulation involves modeling a broader range of behaviour over longer periods of time under controlled conditions, for example, practicing basic life support skills on a training mannequin. Acute simulation focuses on short, critical scenarios and aims to improve responses under stressful conditions; an example of this is a computer model simulating sudden, severe changes in the cardiovascular system following trauma. The acute simulator in use, JustPhysiology, narrows down the extensive model (HumMod) to create a more concentrated interface (14). Another example is Sycamore, an electronic health record-delivered, real-time simulator of patients designed to teach students chronic patient care (14). These products represent a new tool for medical and allied health students to encourage active learning and integrate basic science knowledge into clinical situations (14). Immersion or haptic technology paved the way for a real-time experience of a non-clinical subject (10). Immersive technology encompasses several tools that can be utilized for management, including virtual reality (VR), augmented reality (AR), haptic technology, and telepresence (18). This technology allows a person to feel as if they are present in an immersive environment (virtual environment) (18). These technologies sense the body’s movements, postures, and gestures as input, and these inputs are used to interact with an immersive environment (18). These affect the concepts of the user, space, and human–space interaction. However, the current setbacks to the profound use of such technology are limited, as the focus has been on touch and haptic issues, especially mismatches in places and shapes.

In some cases, these problems appear to stem from reliance on outdated theoretical frameworks (Euclidean-Newtonian). It is suggested that some of the issues could be addressed by referring to Minkowski–Einsteinian space (19).

#### 2.1.2 Integrating simulation with in-person experiential learning: an example of implementation

In line with experiential learning principles, we recommend deploying simulation systems (e.g., JustPhysiology and Sycamore) primarily as adjuncts to face-to-face instruction and laboratory-based kinesthetic activities. Below is a pragmatic lesson plan illustrating how simulation can be interleaved with in-person measurement, small-group reasoning, and structured debriefing in undergraduate medical physiology [BOX 1].

BOX 1 **Example blended session (2–3 h): cardiovascular hemodynamics (adaptable to other systems)**

Pre-class preparation (30–45 min; flipped): Assigned microlecture/reading, a short pre-quiz to prime key concepts (e.g., pressure–flow relationships, compliance, autonomic reflexes), and a brief orientation to the simulator interface.

In-person laboratory session:

1. Readiness assurance and concept check (≈10 min): Audience-response questions or short prompts targeting common misconceptions; instructor clarifies and sets expectations.
2. Kinesthetic measurement station (≈20–25 min): Small groups measure heart rate and blood pressure at baseline and after a simple perturbation (e.g., postural change or light exercise) and document observations.
3. Simulation station (≈25–30 min): Using a cardiovascular simulator, groups manipulate preload, afterload, and contractility, then observe changes in stroke volume, cardiac output, and arterial pressure; they compare simulator outputs to their measured data.
4. Integration worksheet + mini-case (≈20 min): Groups apply their observations to a short clinical vignette (e.g., hemorrhage, dehydration, or heart failure), predict compensatory responses, and justify mechanisms.
5. Facilitated debrief (≈15 min): Structured reflection (what happened, why it happened, and clinical relevance). The instructor links take-home points back to learning objectives.

Post-class consolidation (≈15–20 min): Short formative quiz plus a brief reflection prompt; optional extension scenarios can be completed in the simulator as homework.

Assessment and feedback: Combine low-stakes formative checks (quiz, worksheet, participation) with a brief case-based item in the next tutorial/assessment; collect learner feedback on simulator usability and alignment with laboratory objectives.

### 2.2 Innovations in teaching complex systems

The human body’s complex systems, including the respiratory, cardiovascular, and sympathetic-parasympathetic systems, require distinct perspectives for their application in clinical settings. Virtual reality (VR) enhances physiology education by fostering active learning through interactive engagement, increased interest, improved problem-solving skills, and enhanced feedback (20). Training with AR- and VR-based head-mounted devices (HMDs) was perceived as salient, motivating, and engaging (21). HMDs were effective for certain aspects of medical skills and knowledge learning and training, while other studies suggested that HMDs were only viable as an additional teaching tool, specifically for personalizing education through the application of AI. Studies evaluate the role of AI in personalizing learning by the following criteria: motivation to learn, level of educational outcomes, productivity of communication in the teacher-student system, convenience, and accessibility of education (22). The experimental personalized learning program, developed using artificial intelligence based on student requests, has a positive impact due to its convenience and accessibility, as well as the enhanced productivity of communication in the teacher–student system (22). However, several obstacles exist to the successful adoption and application of AI technology, including user acceptance and striking a balance between AI-assisted and conventional teaching techniques (23). These research hotspots require attention to ensure that the entire technology usage is in the user’s best interest.

### 2.3 Gamification in physiology education

Gamification is emerging as an innovative approach to active learning in medical education, enhancing student engagement and promoting lifelong learning in a unique and collaborative environment. Traditional physiological education predominantly relies on lecture-based teaching, which can be insufficient for effectively engaging students and preparing them for the ever-evolving and collaborative healthcare landscape (24). Furthermore, integration of serious games in medical curricula aligns well with the digital-learning styles of contemporary medical students (25). Few studies have shown the effectiveness of gamification in medical education. A cross-sectional survey conducted in two American universities involving 217 students revealed that 98% of participants favored using technology to enhance healthcare education, while 80% believed video games promote significant educational value (26, 27). A recent study was conducted by introducing the ‘CARBGAME’ (CArd & Board GAMEs) to assess the effect of gamification on cardiovascular physiology teaching. It involved pre- and post-test evaluation of cardiovascular physiology knowledge of 150 students and showed a highly significant improvement. The feedback obtained was extremely positive, particularly in terms of relevance, creativity, engagement, experiential learning, sense of accomplishment, and more (24). Interestingly, a study has also evaluated the impact of gamification on brain activity using an electroencephalogram (EEG). It consisted of a cohort of 14 students, randomly assigned to the gamified (G) and non-gamified (N-G) groups. Analysis revealed higher performance scores in the G group compared to the N-G group and much higher median brainwave activity for attention signals in the G group. Moreover, a positive correlation was established between median attention signals and performance scores (28). Gamification has been shown to significantly enhance student engagement across various educational contexts. Students generally respond positively to gamified learning environments, which can lead to improved academic performance (29, 30).

Thus, gamification in physiology education, through elements like quizzes, leaderboards, and peer competition, significantly enhances student engagement and learning outcomes. Short-term, meaningful gamified interventions are particularly effective, and the approach can be tailored to individual student characteristics to optimize results.

Several innovative approaches are being explored to enhance medical education, including the integration of gamified elements. For instance, a quasi-experimental study by Baashar and colleagues demonstrated the positive impact of an Audience Response System (ARS) on the learning experience of health sciences students (31). Their findings indicated that lectures incorporating ARS led to significantly improved knowledge retention and academic achievement, as well as increased student engagement. Both students and instructors perceived ARS as an easy-to-use and beneficial tool for fostering active learning and providing instant feedback within a traditional lecture setting. Overall, gamification represents a valuable tool for educators aiming to create more interactive and effective learning experiences.

### 2.4 Student-centered learning

Gone are the days when teaching was limited to the board and chalk. In today’s age, the learning landscape has undergone a significant transformation. Nowadays, teachers are equipped with digital tools, and students are more inclined toward concept-based learning rather than rote memorization. One new approach that promotes this type of learning is active learning, which is defined as the activities students engage in to construct knowledge and understanding (32). The study conducted by Ruiz and colleagues identified improvements in failure rates and overall student satisfaction when active learning was adopted (33). Active learning can be carried out in numerous ways, including discussion, group work, and formative assessment. Among these, the “flipped classroom” approach has gained attention in recent years (34). The flipped classroom concept is a very effective learning method and can be considered an alternative to inverting the traditional way of teaching, as suggested by Bishop and Verleger (34, 35). In this model, learning happens at home using online resources and recorded lectures (34, 35). In the classroom, students are actively involved in various tasks that primarily focus on the concepts they have learned at home, which reinforces prior knowledge and allow students to gain practical understanding of how to apply these concepts (36). Another specific type of active learning is problem-based learning. In this model, educators provide a problem to their students, and the students are encouraged to present their unique solutions. This method involves a combination of active learning through brainstorming, group discussions, and presentation skills (37). By providing access to the vast resources made available through digital technologies, students can now present much more refined and specific solutions in a short time (38). Another approach under the active learning spectrum is team-based learning. This method facilitates a structured group discussion, and it involves preparation, problem-solving activities, and testing, along with feedback (39). It encourages the flow of ideas and can result in significantly better outcomes that are widely accepted by the entire class (39). With advancements in technology, team-based learning has improved because it promotes team diversity and exposes group members to a wide range of solutions, enabling them to make informed decisions and obtain better results (39).

### 2.5 Focus on interdisciplinary and integrated curricula

Undergraduate students in medical school study a vast number of subjects, and attempting to grasp the concepts individually can be challenging. However, when they learn related concepts across different subjects, it becomes easier for them to understand and retain information by integrating this knowledge. One of the most fundamental foundations in a student’s medical education is physiology, which primarily focuses on how the human body functions. A study by Narayanan and Merghani focused on teaching the complex concepts of the central nervous system using real-life scenarios (40). The results showed that students exposed to real-life scenarios, formative assessments, and discussions were able to understand the concepts better than their peers who were subjected to traditional learning methods. An approach towards an integrated learning is the systems-based approach (40). It involves the horizontal and vertical integration of concepts, where similar systems are taught across basic sciences and integrated with clinical sciences, respectively (41). For example, a student must have sufficient knowledge of the physiology of the renal system to understand the concept of diuretics (42). The integration of multiple concepts across various fields is possible through collaborative teaching. Studies have shown that collaborative learning development exercises are effective learning tools in medical education. This method supports learning by offering surveys, structured feedback, and analysis of the collaboration between students and teachers (43).

### 2.6 Innovations in assessment of physiological knowledge

For a more accurate assessment of students in medical school, it is beneficial to assess them using adaptive testing through online resources. It enables a reliable and transparent evaluation, allowing the creation of individualized learning modules and support, compared with traditional learning models that expose all students to the same learning modules (44). The newer guidelines in Indian medical education focus on a competency-based medical education, which emphasizes that all students learn observable abilities required in daily practice. This is achieved by integrating clinical and theoretical knowledge and testing students using clinical scenario-based questions (45). To enhance student engagement and foster healthy competition, methods have been employed to integrate lesson objectives into a game-based test series. Studies have shown that students are more engaged, and there is an overall improvement in the grades of the entire class. While the game-based curriculum testing is increasing, it is also important to ensure that it does not deviate from central objectives (46). In physiology, new methods of assessment can involve identifying the affected system and, in turn, understanding the mechanism of that system. This method integrates game-based learning along with the application of knowledge in clinical scenarios.

## 3 Inclusivity and accessibility in teaching medical physiology

Ensuring inclusion and accessibility in medical physiology education is increasingly critical because of the diversity of student needs worldwide. These aspects help ensure that distance, economic, or institutional constraints do not prevent learners from securing quality educational opportunities. This section explores three primary elements, including reducing disparities in resource access, developing Open Educational Resources (OERs), and connecting with remote and under-resourced areas.

### 3.1 Addressing disparities in resource availability

Resource disparity remains one of the most significant challenges facing medical physiology education worldwide, particularly in developing countries and under-resourced areas. Institutions in these areas usually lack updated textbooks, laboratories, or simulation tools for teaching physiology. In the era of digital technologies, this gap can be addressed by enabling learning through virtual classes, labs, and multimedia resources. For example, Al Sayed and colleagues have reported that the Information and Communication Technology facilitates learning by providing easy access to educational resources (47). However, such innovations must be adopted with caution to avoid the depersonalization of learning methods (47). Additionally, digitized learning resources, especially computer-assisted learning, have been found to increase the learning opportunities and improve faculty effectiveness in low and middle-income countries (48).

### 3.2 The advance of open educational resources (OERs)

Open Educational Resources enhance students’ access to high-quality learning materials by alleviating their financial burden. These resources are free and openly licensed, allowing educators to share and adapt them in a manner that best suits different learning environments. Open educational resources, which include open textbooks, lecture videos, and practice materials, are part of a growing repository that is increasingly revolutionizing the way medical physiology education is delivered. A notable initiative in OERs is the DigiWissMed Project, which focuses on developing and disseminating digital competencies among medical students (49). This interdisciplinary approach broadens access, ensuring students receive training tailored to modern medical challenges. Furthermore, collaborative platforms such as MedEdPORTAL and Physiopedia contribute significantly by curating and sharing free resources specific to medical physiology, thereby addressing the global demand for inclusive learning (49).

### 3.3 Engagement strategies for isolated and underserved communities

Special teaching strategies for teaching remote and under-resourced areas are heavily dependent on technologies that reduce the effect of distance and resources. Through the use of virtual classrooms, mobile phone-friendly content, and partnerships with local institutions, medical education has become more accessible to students in remote regions. Online collaborative learning platforms may serve a critical role in providing inclusive learning opportunities for students in distance learning (50). In countries with insufficient infrastructure, virtual labs and real-time online simulations particularly offer a significant advantage (51). Digital proctoring technologies enable students from remote areas to participate in mainstream standardized examinations (51). In addition, efforts by international organizations to provide learning modules in the medical sciences for both health professionals and students in resource-poor settings, as part of WHO e-learning programs, help expand access to educational resources.

## 4 The pedagogical innovations and learning theories

To further contextualize the pedagogical innovations discussed in this review, it is useful to consider them through established learning theory frameworks. In constructivism theory, students learn new concepts through past experiences, interactions, effective communication, and self-reflection. It mainly focuses on active learning models such as inquiry-based learning (IBL), team-based learning (TBL), problem-based learning (PBL), and case-based learning (CBL). It encourages teamwork rather than individual learning (52). The following famous quotation will allow the assumption of constructivism: “The most important factor influencing learning is what the learner already knows” (53). Since this educational theory allows the learners to build upon the new knowledge from past experiences and available interpretive resources, the physiology learning is very much supported by this theory. For example, learning complex concepts such as the cardiac cycle and heart failure in the cardiovascular system, acid–base balance in renal physiology is achieved by using active learning methods such as PBL and CBL. In a recent study, the adapted project-based teaching strategy improved the medical students’ performance in cardiorespiratory and metabolic physiology learning (54). Overall, from a constructivist perspective, many of the emerging approaches in physiology education, particularly those involving AI-supported learning environments, interactive simulations, and case-based activities, facilitate active knowledge construction by enabling students to engage with physiological concepts through inquiry, feedback, and iterative problem solving (55). Cognitive Load Theory also provides a useful lens for understanding how digital tools and AI-assisted platforms can scaffold complex physiological information by segmenting content, providing adaptive explanations, and offering immediate feedback, thereby helping to optimize intrinsic and extraneous cognitive load (55). These adaptive innovations adjust content difficulty according to student performance. It provides chunked digital content in which the physiology topics are broken into smaller, structured modules. The use of interactive diagrams and animations aids in visualizing processes like membrane transport or cardiac cycles, reducing unnecessary cognitive effort. The immediate AI-generated feedback systems help students correct misconceptions before cognitive overload occurs (55). Experiential learning is one of the widely used educational frameworks proposed by Kolb. It is commonly implemented in subjects that involve active learning and the development of practical skills. Although experience plays an important role in this model, students go through a cycle of four phases in which experience is only one component. In this model, students engage in concrete experience, reflective observation, abstract conceptualization, and active experimentation. Throughout this process, students are actively involved in the learning environment and receive continuous feedback that supports cognitive development and modification, ultimately improving student performance (56). This model has a great impact on physiology learning, wherein it bridges theoretical knowledge with hands-on practical learning. Active labs and simulation-based learning are some of the examples of this model (56). In a recent study, it was suggested that laboratory activity integration in the medical degree curriculum enhances the skeletal muscle physiology learning among students (57). Recently, the experiential learning principles have been reflected in the growing use of virtual laboratories, clinical simulation platforms, and AI-driven scenario-based learning, which allow learners to apply theoretical physiological knowledge in authentic or simulated contexts. Together, these theoretical perspectives help explain why technology-enhanced strategies may support deeper conceptual understanding and more meaningful engagement in physiology education (58).

## 5 Challenges in physiology teaching

### 5.1 Barriers and resistance to the establishment of physiology learning

Physiology teaching through innovation, particularly utilizing technology, also faces several challenges and resistances, ranging from barriers to technology integration, faculty training, and balancing traditional and modern approaches, to overcoming organizational resistance. These issues are discussed later in this section.

### 5.2 Barriers to integration of technology in physiology education

**Figure 1:**
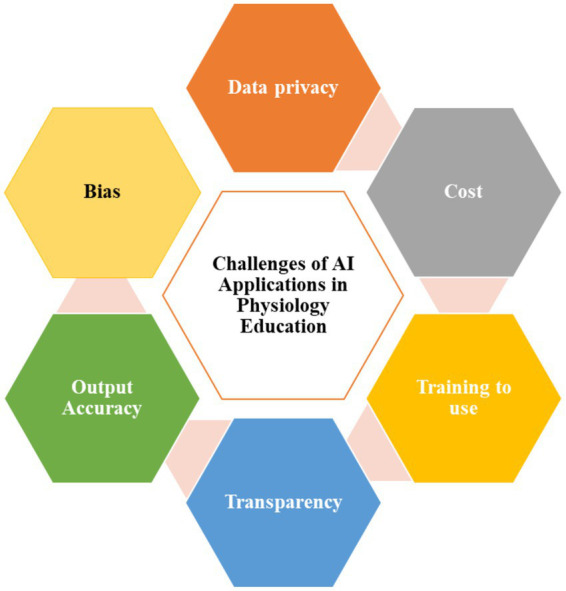
Challenges of AI application in physiology education.

The integration of technology into medical physiology education has the potential to significantly transform current teaching methodologies, but its growth is often restricted due to infrastructure limitations, inadequate training, and resistance to adopting changes among both teachers and learners. According to Jothi and Ponrani, technical difficulties, resource limitations, and a lack of sufficient understanding of the benefits accruing from emerging technologies are the major restrictions (59). The integration of AI and VR in educational contexts is usually resisted on account of high expenses, a lack of readiness for the faculty, and a decrease in self-efficacy regarding computer use (60, 61). These kinds of challenges raise the necessity for accessible resources and developing technological literacy. Figure 1 represents the challenges of AI applications in physiology education.

### 5.3 Training of faculty and utilization of new modalities

Many faculty members are unable to adopt the new modality of teaching due to a shortage of training, limited time, and insufficient institutional support. Additionally, Galikhanov and Khasanova highlighted that educators must first acquire the skills for online teaching (62). Without training, faculty resistance to adopting new methods is one of the greatest barriers (62). Resistance could be due to practical and psychological factors, such as computer anxiety and a lack of confidence, which are among the major factors that hinder the adoption of technology (63). This indicates the need for customized training programs and providing incentives to enhance faculty engagement. Although contemporary technologies and pedagogical methods offer the potential for improved educational outcomes, reconciling these innovations with conventional teaching practices presents a distinct challenge. Many educators and educational institutions tend to favor traditional lecture-based instruction due to its familiarity and perceived effectiveness. This resistance frequently conflicts with the movement toward learner-centered and technology-enhanced methodologies.

## 6 Overcoming organizational resistance

Institutionalized resistance can significantly impede innovation. Most organizational resistance results from insufficient funding, bureaucratic barriers, and the lack of transformative and inspirational leadership. Okunlola emphasizes that effective leadership is essential to overcome systemic barriers and achieve digital transformation (64). Leadership should play a central role in matters of resource allocation, fostering acceptance, and motivating faculty and other staff members. Similarly, Hassall and Lewis argue that the institutional resistance to adopting OER is due to copyright concerns, lack of motivation, and workflow disruptions. They also suggest that these challenges can be addressed through sound policy, targeted training, and appropriate incentives (65).

## 7 Future directions in physiology teaching

Physiology education is at a crossroads, requiring adjustments in both methodologies and tools to meet contemporary demands. The probable future may focus on the integration of sophisticated technologies, holistic learning environments, and a shift toward transformative pedagogies over the next decade.

### 7.1 Artificial intelligence/machine learning

**Figure 2:**
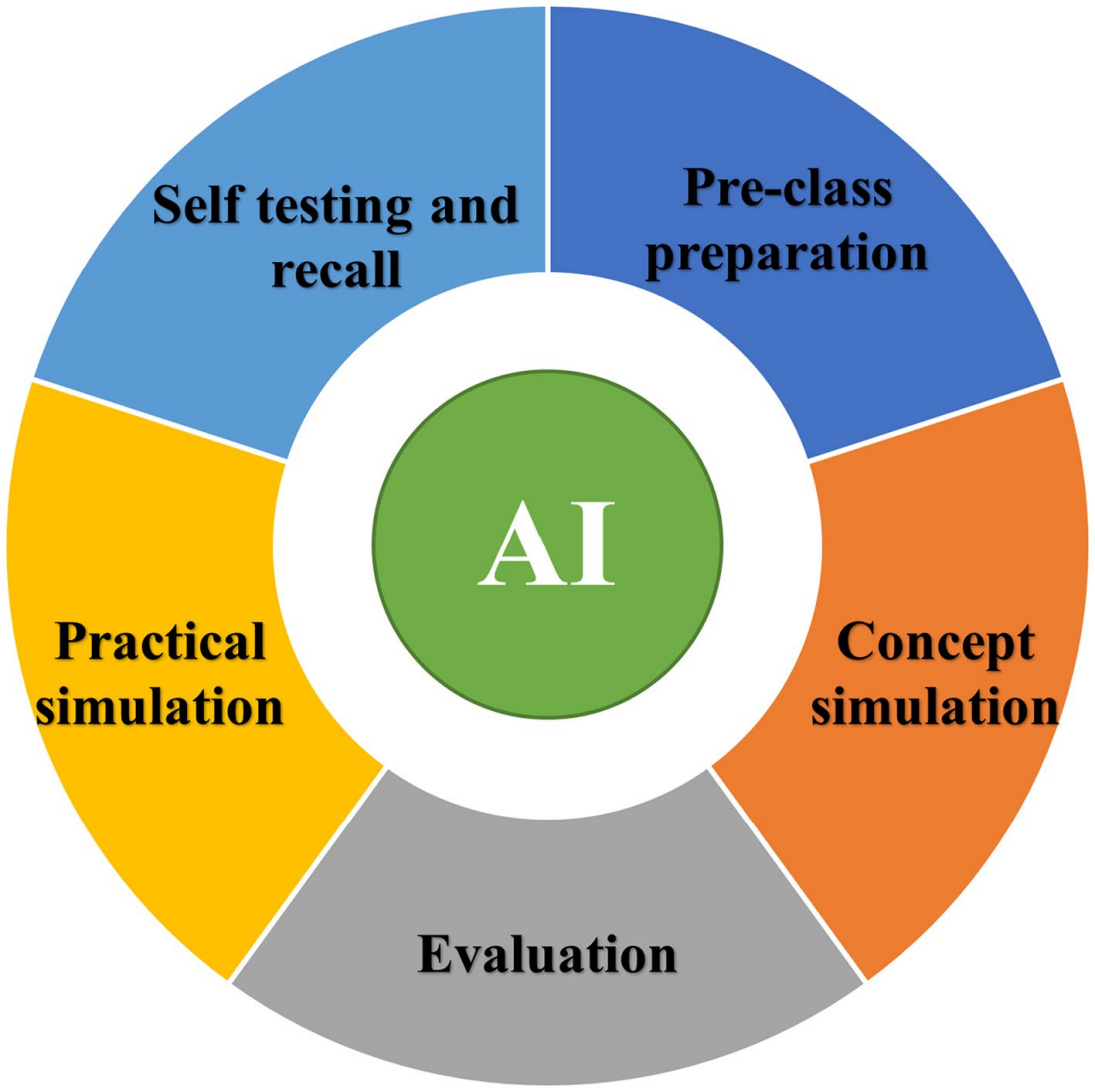
AI’s possible applications in physiology education.

AI has started to be integrated in medical education, starting from early pre-clinical years, such as anatomy, to its deployment in teaching and diagnosing advanced clinical specialties such as neurology and neurosurgery (66, 67). The integration of AI and machine learning into physiology education aims to enable more dynamic and individualized learning. Virtual patients and intelligent tutoring systems are AI tools that enhance the clinical context of physiology education by simulating real-life circumstances. The Federation of European Physiological Societies (FEPS) pays close attention to these technologies in its effort to modernize physiology education across Europe (68). AI platforms can examine the learning styles of individual students to detect specific problems and provide solutions to improve those areas. As some studies argue, the combination of problem-based learning (PBL) and AI tools is useful in ensuring a deep understanding of medical concepts and improving reasoning and problem-solving skills (69). The generative AI tools like ChatGPT-4 can facilitate interactive learning by generating questions for practice. However, their integration may introduce challenges in a few areas, particularly the assessment integrity (70). Apart from tutoring systems, AI-enhanced platforms have been incorporated into a variety of online education platforms to enhance immersive and data-driven learning. Simulations of real-time physiological processes in the human body are provided by PhysioNet and Visible Body through AI-powered simulations, which help in visualizing very abstract concepts better. Moreover, BioDigital Human, a comprehensive anatomy and physiology platform, uses AI to offer customized adaptive learning based on the achievements of students (71, 72). AI interdisciplinary virtual labs make it possible to transform theories into practice. Some platforms, such as Labster, utilize AI to reproduce complicated physiological experiments, enabling learners to test their assumptions and “see” the biological phenomena without real-life risks and costs (73). This approach is especially important for those institutions that do not have enough funds for physical laboratories, making the study of physiology more widely available around the world (74). AI tools are also effective in medical physiology education for understanding the pathophysiological mechanisms underlying disease development. For example, the role of protein synthesis in the aqueous humor can be investigated using single-cell transcriptomics integrated with proteomics from liquid biopsies (75). Additionally, the Dandelion algorithm is used for training interspecies Bayesian networks in gene regulatory network research (76). AI is being utilized to assist with progress tracking and grade compilation, where it provides instant grading and performance analytics for students and teachers. Google Socratic and Cerego, among others, use AI algorithms, and they adapt according to how the learner previously answered for maximum accuracy. However, scientific evidence demonstrating the role of these tools has yet to be evaluated. Despite the development of numerous AI tools in medical education, studies exploring evidence of their effectiveness in physiology education remain scarce (72). Figure 2 illustrates the potential applications of AI in physiology education.

The use of technology in teaching physiology, combined with AI and machine learning, is not merely about saving time; it is also about personalizing learning to make it user-friendly, engaging, and student-centered. By comprehensively examining diverse learning patterns and streamlining teaching strategies, AI in medicine is transforming the way physiology is taught and learned today, propelling us toward a better, more customized, and efficient medical education system.

### 7.2 Development of holistic learning environments

The holistic learning ecosystems integrate various learning resources, approaches, and environments into a unified learning experience. The concept put into physiology education encompasses everything from traditional lectures and interactive digital resources to interdisciplinary collaboration. The examples for physiology education range from a central concept of integrating major ideas from multiple disciplines, which would drive physiology education away from being a content-driven curriculum toward a deeper understanding (77). There is an increasing need for physiology teaching to be integrated within the clinical sciences. Clinically Oriented Physiology Teaching (COPT) integrates case studies and clinically applied physiology into conventional physiology courses, resulting in significant improvements in student performance and satisfaction (78). Holistic ecosystems also extend beyond the classroom. For example, interdisciplinary approaches, such as collaboration between universities and basic education institutions, promote health education and physiology awareness in broader communities (79).

### 7.3 Vision for the next decade

Over the coming decade, physiology teaching is likely to become more integrated into clinical learning, facilitated by the advancement of virtual and augmented reality. This is likely to be accompanied by further development of interactive tools that model human anatomy and physiology processes, bridging the gap between concept and clinical application (80). The focus on active and learner-centric pedagogies is expected to intensify, underpinned by principles derived from cognitive psychology and teaching methodologies grounded in empirical evidence. This educational framework aspires to enable students to develop into autonomous learners who can adeptly manage intricate medical dilemmas (74). Furthermore, through FEPS, international efforts are being made to harmonize educationally active standards, embed biomedical sciences within physical sciences, and facilitate lifelong learning facilities (68). To synthesize the innovations discussed in this review, we propose an integrative conceptual framework in which technological tools (e.g., AI, simulations, and gamification, digital learning platforms, and virtual laboratories) interact with pedagogical approaches (e.g., student-centered and interdisciplinary learning, active learning, simulation-based learning, and personalized instruction) and equity-focused strategies (e.g., open educational resources and initiatives for underserved communities) to create an adaptive learning ecosystem. Together, these elements collectively reshape physiology education by enhancing engagement, supporting experiential learning, and promoting equitable access. Additionally, this framework highlights how emerging technologies function not as isolated tools but as components of an integrated ecosystem that supports personalized, data-informed, and experiential learning in physiology.

Several limitations must be addressed when considering the balanced integration of technology-based tools and methods alongside traditional teaching approaches. Few studies have explored the effectiveness of digital learning platforms or active learning methods specifically in physiology education. Each emerging technological tool should undergo proper validation prior to implementation. Overuse or recommendation of excessive tools or methods may negatively impact student engagement and the overall learning environment. Furthermore, improper integration of these tools could compromise foundational learning. Evidence regarding the long-term benefits of tools such as simulation-based learning remains limited.

## 8 Conclusion

Medical physiology teaching has evolved significantly, transitioning from traditional lectures and textbooks to a more dynamic and technology-driven approach. This review demonstrates how modern tools, including VR, AI, simulation-based learning, and gamification, are transforming the learning process in medical physiology education. These advanced tools not only enhance the interactivity of learning but also enable students to develop critical thinking skills and apply their knowledge in real-world clinical settings. Simulation-based education, for example, allows students to practice in lifelike scenarios without the risks associated with real-world mistakes. The digital education platforms, such as Just Physiology and Sycamore, engage students by having interactive simulations, virtual experiments, and complex patient cases. On the other hand, VR and AR further enhanced the learning process by allowing students to explore 3D models and visualize physiological processes in action, making complex concepts easier to understand. Gamification is another powerful tool, incorporating elements such as quizzes, leaderboards, and peer competition to keep students motivated. It makes learning fun while also boosting engagement and retention. Recent innovations such as CArd and Board Games, CARBGAME, are shown to be effective in improving the learner focus and cognitive performance. Ongoing developments in AI tools are having a huge impact on personalizing the learning experience. With adaptive quizzes, virtual patients, and intelligent tutoring systems, AI tailors content to each student’s needs. Platforms like PhysioNet and BioDigital Human can even simulate real-time physiological processes, making abstract concepts more concrete. This level of customization helps students learn more efficiently and stay engaged.

Despite having various advances in technology and resources, there are still several challenges. These include disparities in resources, resistance to tech adoption, and the need for better faculty training. Additionally, privacy and ethical concerns around the use of AI are still debatable in medical education. After careful consideration of these challenges, focusing on the integration of learning resources, tech in classrooms, and conducting comprehensive training programs for teachers would help in advancing the modern physiology education to a high standard. Continuous feedback between tutors and students on these emerging technologies, along with appropriate modifications, will enhance the student-centered learning experience in a contemporary physiology education. Furthermore, adopting digital technologies along with self-regulated learning as a multidisciplinary approach would be the most effective strategy for promoting both theoretical and practical skills. Future research should focus on exploring the practical impact of each technology-based tool or method to further expand the use of such in the physiology education.
